# Fifty Years of Coronary Artery Bypass Graft Surgery

**DOI:** 10.21470/1678-9741-2017-0104

**Published:** 2017

**Authors:** Eduardo Augusto Victor Rocha

**Affiliations:** 1 Hospital Universitário Ciências Médicas de Minas Gerais (HUCM), Belo Horizonte, MG, Brazil; 2 Faculdade de Saúde e Ecologia Humana (FASEH), Belo Horizonte, MG, Brazil

On May 9, 1967, 50 years ago, Dr. René G. Favaloro, an Argentine surgeon operating at the Cleveland Clinic, officially performed the first coronary artery bypass graft surgery (CABG) of the modern era, historically, with many contributions. Alexis Carrel, in 1910, performed the first experience in dogs, using carotid anastomosis with the left coronary artery^[1]^. However, he discouraged other surgeons to perform this procedure, because he believed the anastomosis should be performed in less than three minutes. In the 1940's and 1950's, other surgeons studied myocardial revascularization, such as Gordon Murray, in Toronto, and Vladimir Demikhov, in Moscow. In the 1960s, although several groups were studying revascularization, Michael DeBakey did not believe in its success and did not think it should be performed in humans.

Robert Goetz was the one who performed and published the first coronary artery bypass graft surgery in humans in 1961, using a tantalum ring^[2-6]^. Arthur Vineberg^[7]^ published, in 1962, on the implantation of the internal thoracic artery in the myocardium, with other previous studies by this author. Vasilii Kolesov^[2]^ published his Russian experience in 1965^[2]^ and a series of 12 cases in the Journal of Thoracic Surgery in October 1967^[8]^. William Longmire published in French in 1966. Other authors such as Donald Kahn, Edward Garrett and David Sabiston did not publish until 1971, 1973 and 1974, respectively.

Favaloro^[3]^ performed the first saphenous vein anastomosis on May 9, 1967, with another 13 cases until October, publishing his results in April 1968. Although the paternity of this procedure is debatable, Favaloro marks the beginning of CABG of the modern era. Several surgeons continued to contribute to the development of this technique, among these some notable Brazilians. Surgery without cardiopulmonary bypass (CPB), conceived by Dr. Enio Buffolo^[6]^, is one such contribution. Dr. Ricardo Lima^[9]^ was also relevant for performing the revascularization of the posterior branches of the circumflex artery without CPB. The use of dual mammary artery, radial artery, minimally invasive procedures are some of the advances incorporated into classic CABG.

CABG is probably the most life-saving surgery in the world. Favaloro had a tragic end of life. Frustrated with the financial difficulties faced by his foundation in Argentina, he gave up his life in 2000. A few years ago with the advent of coronary angioplasty and the development of stents, the probable extinction of CABG was decreed. However, trials such as Synthax, Freedom, Nobel, EXCEL among others demonstrate their efficiency with consistent results. As long as no scientific evidence of any better therapy than CABG was found, we will keep on operating and remembering the above-mentioned pioneers. Fifty years later, CABG is more alive than ever showing its strength.

## Figures and Tables

**Table 1:** The First Clinical Coronary Artery Bypass Operations

Dale	Surgeon	Graft	Technique	Follow-Up
May 2,1960	Goetz	RITA	Tantalum ring	No angina at 1 year
				Pt died of AMI 15 years later
April 4, 1962	Sabiston	SV	Suture	Pt. died 3 days later
				(This case first reported in 1974)
Feb 25, 1964	Kolesov	LITA	Suture	No angina at 3 years' follow-up
Nov 23, 1964	Garrett	SV	Suture	No angina at 7 years' follow-up
	Dennis			(This case first reported in 1973)
	DeBakey			
March 22, 1967	Kolesov	LITA	Stapling	No angina at 3 years' follow-up
May 9, 1967	Favaloro	SV	Suture	Successful
Feb 29, 1968	Green	LITA	Suture	Successful

Source: Konstantinov et al.^[5]^ . AMI = acute myocardial infarction; LITA = left internal thoracic artery; RITA = right internal thoracic artery; SV = saphenous vein.
